# Spatial Control of Cell-Nanosurface Interactions by Tantalum Oxide Nanodots for Improved Implant Geometry

**DOI:** 10.1371/journal.pone.0158425

**Published:** 2016-06-30

**Authors:** Udesh Dhawan, Hsu An Pan, Chia Hui Lee, Ying Hao Chu, Guewha Steven Huang, Yan Ren Lin, Wen Liang Chen

**Affiliations:** 1 Department of Materials Science and Engineering, National Chiao Tung University, Hsinchu, Taiwan, ROC; 2 Hokan Life Technology, Taichung, Taiwan, ROC; 3 Department of Emergency Medicine, Changhua Christian Hospital, Changhua, Taiwan, School of Medicine, Kaohsiung Medical University, Kaohsiung, Taiwan, School of Medicine, Chung Shan Medical University, Taichung, Taiwan, ROC; 4 Department of Biological Science and Technology, National Chiao Tung University, Hsinchu, Taiwan, ROC; Texas A&M University Baylor College of Dentistry, UNITED STATES

## Abstract

Nanotopological cues can be exploited to understand the nature of interactions between cells and their microenvironment to generate superior implant geometries. Nanosurface parameters which modulate the cell behavior and characteristics such as focal adhesions, cell morphology are not clearly understood. Here, we studied the role of different nanotopographic dimensions in modulating the cell behavior, characteristics and ultimately the cell fate and accordingly, a methodology to improve implant surface geometry is proposed. Tantalum oxide nanodots of 50, 100nm dot diameter with an inter-dot spacing of 20, 70nm and heights 40, 100nm respectively, were engineered on Silicon substrates. MG63 cells were cultured for 72 hours and the modulation in morphology, focal adhesions, cell extensible area, cell viability, transcription factors and genes responsible for bone protein secretion as a function of the nanodot diameter, inter-dot distance and nanodot height were evaluated. Nanodots of 50nm diameter with a 20nm inter-dot spacing and 40nm height enhanced cell spreading area by 40%, promoted cell viability by 70% and upregulated transcription factors and genes twice as much, as compared to the 100nm nanodots with 70nm inter-dot spacing and 100nm height. Favorable interactions between cells and all dimensions of 50nm nanodot diameter were observed, determined with Scanning electron microscopy and Immunofluorescence staining. Nanodot height played a vital role in controlling the cell fate. Dimensions of nanodot features which triggered a transition in cell characteristics or behavior was also defined through statistical analysis. The findings of this study provide insights in the parameters of nanotopographic features which can vitally control the cell fate and should therefore be taken into account when designing implant geometries.

## Introduction

Nano-topography can modulate cell behavior [1], cell morphology [2], cell proliferation [3], cell migration [4], cell physiology [5] and ultimately, the cell fate [6]. The size as well as the shape of the nano-topographies like nano-dots [4, 6–8], nano-islands [9], nano-concave [10], nano-grooves [11–14], nano-tubes [15], nano-ridges [16, 17], and nano-pores [18] have been seen to act as stimuli to affect and guide the cellular response. In addition, roughness of the nano-surface has also been seen to modulate cell response such as cellular adhesion [19]. 2D nano-surfaces made from Titanium [20] as well as 3D surfaces [21] have also been seen to modulate cell behavior. Several materials such as Silicates [22], Titanium [23], and Tantalum oxide [5] have been exploited in the field of Biomedical Engineering due to their extraordinary biocompatibilities. A plethora of studies have been done in the past to elucidate the effect of variation in size of Tantalum oxide nano-dots on cellular behavior [24]. Osteoblasts [3], NIH-3T3 cells [8], cardiomyocytes [6] as well as several cancer cells such as C33A, TOV-112D, TOV-21G have been seen to react to nano-dots of different sizes by displaying different morphologies as well as modulation in cell characteristics such as focal adhesions, microfilament bundles, cell area. All of these studies collectively validate the effects of the nano-topographies on the cellular behavior. However, in-vivo, the tissue microenvironment regulates the cell behavior and vice-versa [25, 26]. Cells have continuous physical [26] and biochemical interactions with their microenvironment and any change in this microenvironment can directly or indirectly control the cell fate.

Tissue microenvironment displays a highly diverse stroma consisting of extracellular Matrix (ECM,) homotypic or heterotypic population of cells, and nano/microscale topography [27]. Physically, tissue microenvironment displays a highly structured architecture. However, physical as well as biochemical changes in this microenvironment can modulate the cell parameters such as cell morphology, cell adhesion etc. Physical changes such as change in the matrix stiffness can trigger intracellular signaling cascades within the cell which may also affect the normal cellular functioning. The composition as well as the properties of the tissue microenvironment are crucial for cellular function and any variation can have a profound effect on its constituents. This implies that homogeneity of the tissue microenvironment is of utmost concern. However, since many of the tissue microenvironment’s constituents lie in the nano-range, it is possible that highly homogeneous nano-topographies may be able to compliment tissue microenvironment’s architecture, which can be exploited to study the interactions between the cells and their microenvironment and elucidate why some nano-topographies offer more favorable interactions than the rest.

Even though multiple attempts have been made by the researchers in the past to discover new materials that provide favorable environment to the cells for their growth and to be used as implants, an interesting question which remains unanswered is why cells respond differently to different nanomaterials. We have shown in our previous study on osteoblast-like cells that nano-topography modulates not only the cell morphology but also affects the degree of mineralization [3]. Other studies pertaining to osteoblasts have proved that the surface topography also modulates the amount of bone deposited adjacent to the implant [28]. However, the reason behind this modulation, the nature of interactions between the cells and the nano-surface and why a nano-surface with a specific topography triggers a particular response from the cells than the rest is still unclear. Thus, it is of utmost importance to elucidate these interactions and the reasons behind these selective behaviors. Unravelling these reasons can lead to engineering of improved nano-surface geometries which enhance cellular response and can be used as implants. Besides, even though multiple materials with extraordinary biocompatibilities exist, however, the nature of interactions at the material-biological entity interface and the size of the nano-structures on the material which induce transition in the cell characteristics also needs to be explored and defined.

The present study elucidates the role of different nanotopography dimensions in modulating the cell behavior, characteristics and ultimately the cell fate. Two different nanotopographies, one with nanodot diameter of 50nm, inter-dot spacing of 20nm and nanodot height of 40nm and another with nanodot diameter of 100nm, inter-dot spacing of 70nm and nanodot height of 100nm, were engineered out of Tantalum oxide by using aluminum pores as template to elucidate the role of different nanotopography features in modulating cellular response. MG63, osteoblast-like cell line, isolated from human osteosarcoma was chosen as a model for this study. Modulation of cell characteristics such as cell morphology, focal adhesions, cell viability and transcription factors, genes coding for bone proteins in response to the different nanotopography parameters (nano-dot diameter and inter-dot distance and nanodot height) was studied which may assist biomedical engineers in designing implants with geometries for an improved cell response. The findings of this study may find applications in the fields of Biomedical and tissue engineering.

## Materials and Methods

### Chemicals

Glutaraldehyde and osmium tetroxide were purchased from Electron Microscopy Sciences (USA). Anti-vinculin rabbit polyclonal antibody (catalogue BS-6640R) was purchased from Bioss (USA). Alexa Fluor 594 phalloidin (catalog A12381) was purchased from Thermo Fisher Scientific (USA) and Alexa Fluor 488 FITC conjugated goat anti-rabbit antibody was purchased from Jackson Immunoresearch (USA). Trypsin was purchased from Sigma (USA). Bovine serum albumin (BSA) was purchased from GIBCO (Thermo Fisher Scientific Inc. USA). Collagen I and Fibronectin were purchased from Sigma Aldrich (U.S.A.). Phosphate buffered saline (PBS) was purchased from Bio-tech (Taipei, Taiwan). Sulfuric acid (H_2_SO_4_), oxalic acid (H_2_C_2_O_4_), and phosphoric acid (H_3_PO_4_) were purchased from Sigma Chemicals (Perth, Western Australia), 6-inch silicon wafers, Aluminum ingots were purchased from Admat-Midas (Norristown, PA, USA). Paraformaldehyde, Triton X-100 were purchased from Alfa Aesar (Taiwan). Other chemicals of analytical grade or higher were purchased from Sigma or Merck (USA).

### Fabrication of Tantalum oxide nanodots

Tantalum oxide nano-dots were fabricated on top of Silicon substrates. A 200nm thick film of Tantalum Nitride (TaN) was first sputtered onto a 6-inch silicon wafer (Summit-Tech, West Hartford, CT, USA), followed by the deposition of 400nm thick film of Aluminum using a thermal coater. Anodization was carried out in 0.3 M oxalic acid at 25 V for 90 min for the 50nm. 100nm nano-dot arrays were fabricated by a two-step anodization method. In the first anodic oxidation step, anodization was carried out in 0.3 M oxalic acid at 50 Volts for 10 min. The porous alumina was removed by immersing in 5% (w/v) H_3_PO_4_ for 70 minutes. Second anodization step is repeated in the same way as the first one. Porous anodic alumina was formed during the anodic oxidation. The porous alumina was removed by immersing in 5% (w/v) H_3_PO_4_ overnight. In the first step, the top-most aluminum layer gets oxidized to alumina followed by the outward diffusion of Al^3+^ and inward diffusion of O^2-^ due to the applied electric field. This leads to the formation of the vertical channels in the aluminum layer. The depth of the channels increases with time. The dissolution of alumina at the alumina/electrolyte interface stays in equilibrium with growth of the vertical channel in the aluminum layer. Finally, as the alumina layers reaches the TaN/Aluminum interface, oxygen is continuously injected into the TaN layer which gets oxidized to Ta_2_O_5_^.^ This results in the volume expansion of the Tantalum which gives rise to the hemispherical structures (Tantalum oxide nano-dots). This extent of volume expansion of Ta is directly proportional to the voltage applied for the electrolysis and the duration. Hence, the dimensions of the Tantalum oxide nano-dots can be controlled directly by controlling the Voltage and time. The dimensions and homogeneity of nanodot arrays were measured and calculated from images taken by JEOL JSM-6500 TFE-SEM and summarized in Table 1. A thin layer of platinum was sputtered onto the structures.

**Table 1:** List of fabricated nanotopographies with corresponding features.

Nanodot Diameter (nm)	Spacing (nm)	Height (nm)
Flat (0nm)	0nm	0nm
50nm	20nm	40nm
100nm	70nm	100nm

### Cell culture

MG63 osteoblast-like cells, used for the experiments in this study were originally isolated from human osteosarcoma (BCRC no. 60279, Bioresources Collection and Research Center, Taiwan). MG63 cells were cultured in Eagle’s minimum essential medium with 2 mM L-Gutamine and Earle’s BSS adjusted to contain 1.5g l^-1^ sodium bicarbonate, 0.1mM non-essential amino acids and 1.0 mM sodium pyruvate. Eagle’s minimum essential medium was supplemented with 10% fetal bovine serum (FBS, GIBCO Invitrogen). Cells were incubated at 37°C in 5% CO_2_ atmosphere. To eliminate any possible contamination of nano/micro particles, cell culturing was performed in a class-10 clean room.

### Morphological analysis using Scanning Electron Microscopy

MG63 cells were seeded on the nanodots with a different dot diameter and inter-dot distance. After removing the culture medium, the wells were rinsed with Dulbecco’s Phosphate Buffered Saline (DPBS). The cells were fixed with 1.25% gluteraldehyde in PBS at room temperature for 15 minutes, followed by staining with 1% osmium tetraoxide for 30 minutes. Samples were then washed with PBS 3 times, 5 minutes each and finally immersed in 40% alcohol overnight. Dehydration was performed the next day using a series of ethanol concentrations (10 min incubation each in 50%, 60%, 70%, 80%, 90%, 95%, 100% ethanol). The samples were sputter-coated with Platinum and examined by using by JEOL JSM-6500 TFE-SEM at an accelerating voltage of 8 Kiloelectron volts (KeV).

### Immunostaining of Actin filaments and vinculin

Cells were harvested and fixed using 4% paraformaldehyde in PBS for 15 min followed by 5 PBS washes. Cell membranes were permeabilized using 0.1% Triton X- 100 incubation for 15 min, followed by 5 washes in PBS. The membranes were then blocked overnight using 2% BSA in PBS followed by 5 PBS washes, the next day. The samples were incubated with Mouse anti-Human anti-vinculin antibody (1:100, diluted in 2% BSA) and phalloidin (working concentration of 6.6 μM) for 1 h, followed by incubating with Alexa Fluor 488 goat anti-mouse antibody (1:100) for 1 h and followed by three PBS washes. Samples were mounted and imaged using a Leica TCS SP2 confocal microscope. All PBS washes were performed for 5 minutes each.

### Coating of Tantalum oxide nanodots with proteins

Proteins, namely, BSA, Collagen, and Fibronectin were prepared in the liquid form with the final concentration of 1%. Proteins were pipetted on-to the nanosurfaces until the surface was completely covered and left to dry for 2 hours in the laminar air flow hood to avoid any chances of contamination. Finally, protein coated nanodots were washed thrice with PBS and used for the experiments.

### Treatment of cells with Cytochalasin D

Cells were grown on nanodots with different dot diameters (50nm, 100nm), inter-dot spacing (20, 70nm) and nanodot heights (40, 120nm). TaN coated Si substrates were taken as controls. On the final day, cells were subjected to the medium containing 10μM Cytochalasin D for 60 minutes at 37 degrees Celsius and 5% CO_2_. After 60 minutes, cells were washed twice with PBS, P.H. 7.4. For analysis using SEM, cells were fixed with 1.25 Glutaraldehyde for 15 minutes at room temperature followed by staining with Osmium tetroxide for 30 minutes followed by 3 PBS washes and finally immersed in 40% alcohol overnight. The next day, samples were dehydrated using a series of ethanol concentrations (10 min incubation each in 50%, 60%, 70%, 80%, 90%, 95%, 100% ethanol) and analyzed using SEM. For analysis using confocal microscope, the protocol previously mentioned (section 5.5) was followed.

### Quantitative real-time PCR

Quantitative Real time PCR was performed as described previously. In brief, q-PCR was performed to investigate the nanotopographic effects on the gene expression level. Oligo primers of transcription factors Osterix, RUNX and genes coding for Osteocalcin, Osteopontin, Bone Sialoprotein, Collagen, alkaline phosphatase and β–Actin are listed in the Table 2. TRIreagent (Talron Biotech) was used to extract total RNA and purified using an RNeasy Mini Kit (Qiagen). Then, RNA was suspended in DEPC-treated water and quantified at OD260. cDNA was synthesized by annealing 29ul of total RNA by using 1ug of oligo-dT primer. Reverse transcription was performed by using SuperScript-III Reverse Transcriptase (Invitrogen) on an iCycler iQ5 (Bio-Rad Laboratories). The following cycling conditions were used; 1 cycle, 5 minutes at 95°C; 50 cycles, 20 seconds at 95°C; 20 seconds at 55°C; 40 seconds at 72°C. Threshold cycles, (Ct), determined by the iCycler iQ Detection System software were used to get the expression levels. ΔΔCt method was used to get the Relative transcript quantities. β-Actin was used as a reference gene. ΔCt was defined as the difference in the threshold cycles of target mRNA relative to β-Actin mRNA. ΔΔCt was defined as the difference between ΔCt of cells grown on the controls and ΔCt of cells cultured on the different nanodots. 2ΔΔCt was defined as the fold change in the mRNA expression. The results were expressed as the mean SD of six experiments.

**Table 2:** Forward and Reverse primers of transcription factors and Genes used for qRT-PCR.

Transcription factor/Gene	Forward	Reverse
**Osterix**	5-agttcacctgcctgctctg-3	5-tctgactggcctcctcttc-3
**Bone Sialoprotein (BSP)**	5-ccaccaccgttgaatacgag-3	5-tagccatcgtagccttgtcc-3
**Osteocalcin**	5-ggcagcgaggtagtgaaga-3	5-tgtggtcagccaactcgtc-3
**Osteopontin**	5-cccttccaagtaagtccaacgaaagc-3	5-ctggatgtcaggtctgcgaaacttc-3
**RUNX2**	5-tctggccttccactctcagt-3	5-tatggagtgctgctggtctg-3
**Collagen type I**	5-atccgcagtggcctcctaat-3	5-tcccctcaccctcccagtat-3
**β-Actin**	5-atgggtcagaaggattcctatgtg-3	5-gccagattttctctccatgtcgtc-3
**Alkaline Phosphatase**	5-ttgtgccagagaaagagagaga-3	5-gtttcagggcatttttcaaggt-3

### Cell Viability Assay

Cells were cultured for 72 hours on the nanodots of different sizes and inter-dot spacings. After 72 hours, medium was decanted and cells were washed twice with dPBS and incubated in Hoechst stain, H33342, which binds to the AT-rich regions of double stranded DNA. Number of stained cells were counted on the different nanosurfaces, normalized against control surfaces (Flat Silicon) and expressed as the percentage of viable cells.

### Statistics

All experiments were performed in triplicates. Data were expressed as the mean and standard deviation. One-way analysis of variance followed by a Tukey post-test was used for statistical analysis (SPSS 13.0 software, Chicago, SA), and the level of significance was set at P<0.05.

## Results

### Fabrication of Tantalum oxide nanodots

**Fig 1:**
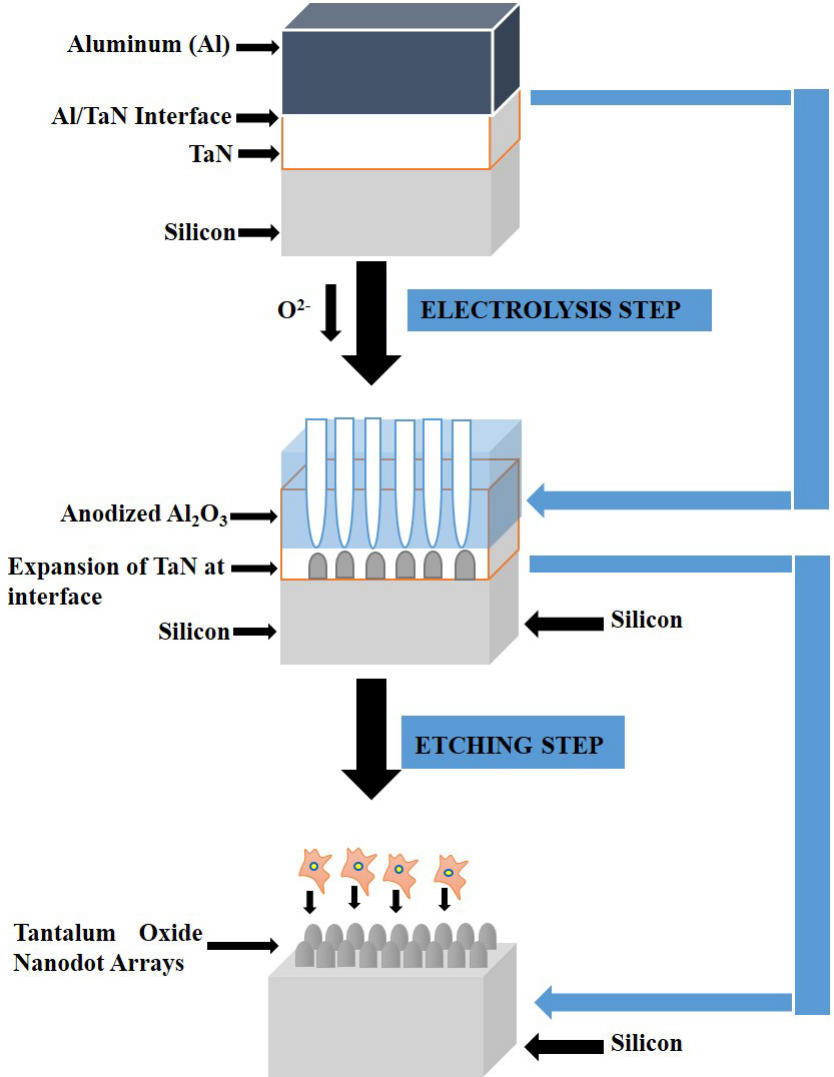
Schematic representation of fabrication of Tantalum oxide nanodot arrays.

**Fig 2:**
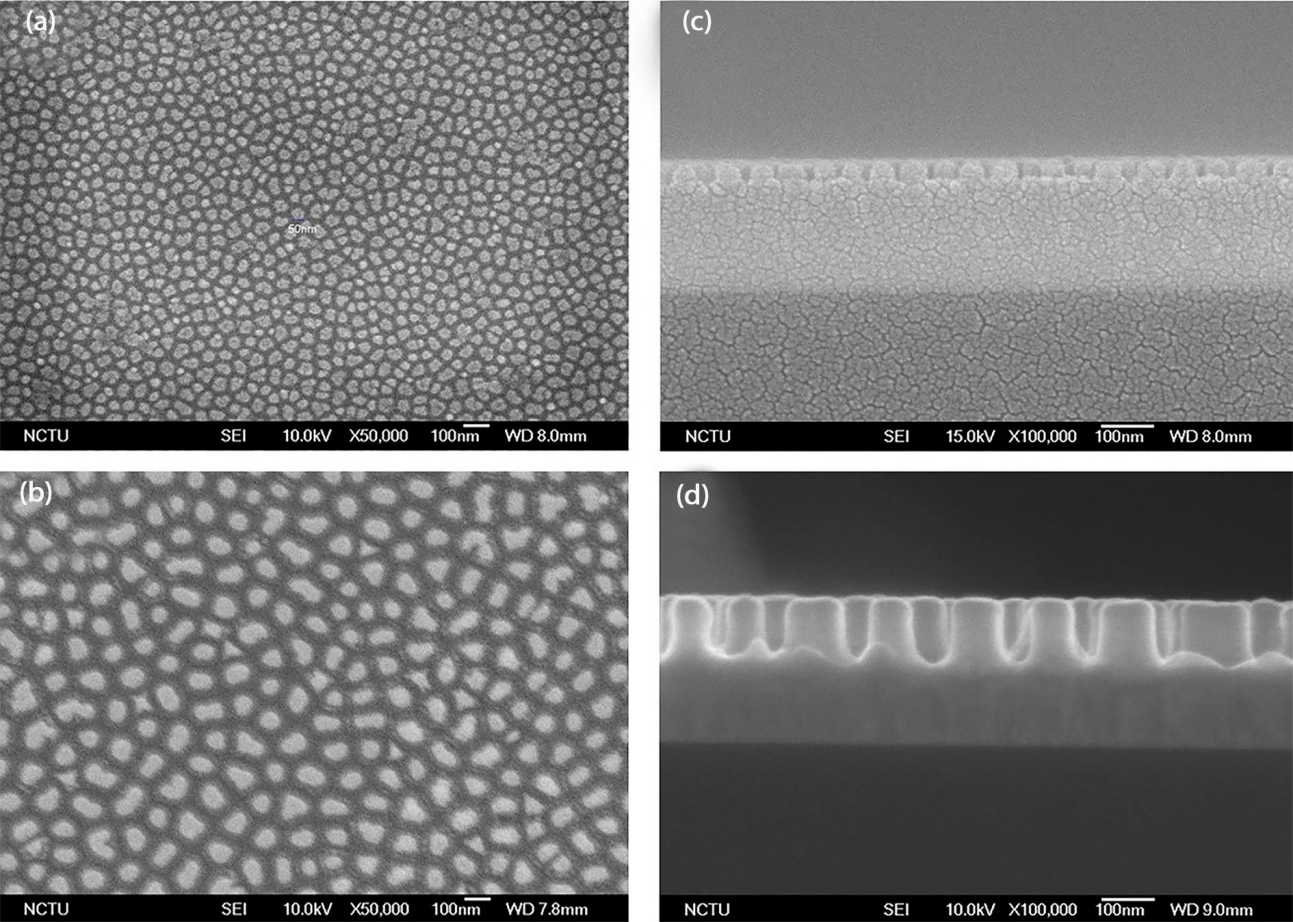
Scanning electron microscopy of Tantalum oxide nanodots. (a), (c): Top and cross-sectional view of nanodots with 50nm diameter, 20nm inter-dot spacing and 40nm nanodot height. (b), (d): Top and cross-sectional view of nanodots with 100nm diameter, 70nm inter-dot spacing and 100nm nanodot height. Scale bar = 100nm.

Homogeneous Tantalum oxide nano-dots with 50, 100nm dot diameter were fabricated by Anodic Aluminum oxide (AAO) processing on the Aluminum-Tantalum Nitride coated Silicon Wafers. Schematic representation is shown in Fig 1. TaN coated Silicon substrates were used as control. The dimensions of nanodots were well controlled and highly defined. The nanodots’ diameters of 50.35 ± 3.2 and 99.4 ± 6.3 and inter-dot spacing of 20.37 ± 1.85nm and 70 ± 7.4nm and nanodot heights 38.89 ± 3.7nm and 100.5 ± 2.6nm respectively were observed after analyzing with Scanning electron microscope (JEOL JSM-6500 TFE-SEM). Top, as shown in Fig 2A and 2B as well as cross-sectional imaging, as shown in Fig 2C and 2D was done to analyze the diameter, spacing between two successive nano-dots as well as nanodot heights.

### Nanodots with different dot diameter, inter-dot spacing and nanodot heights modulated cell morphology and the number of focal adhesions

**Fig 3:**
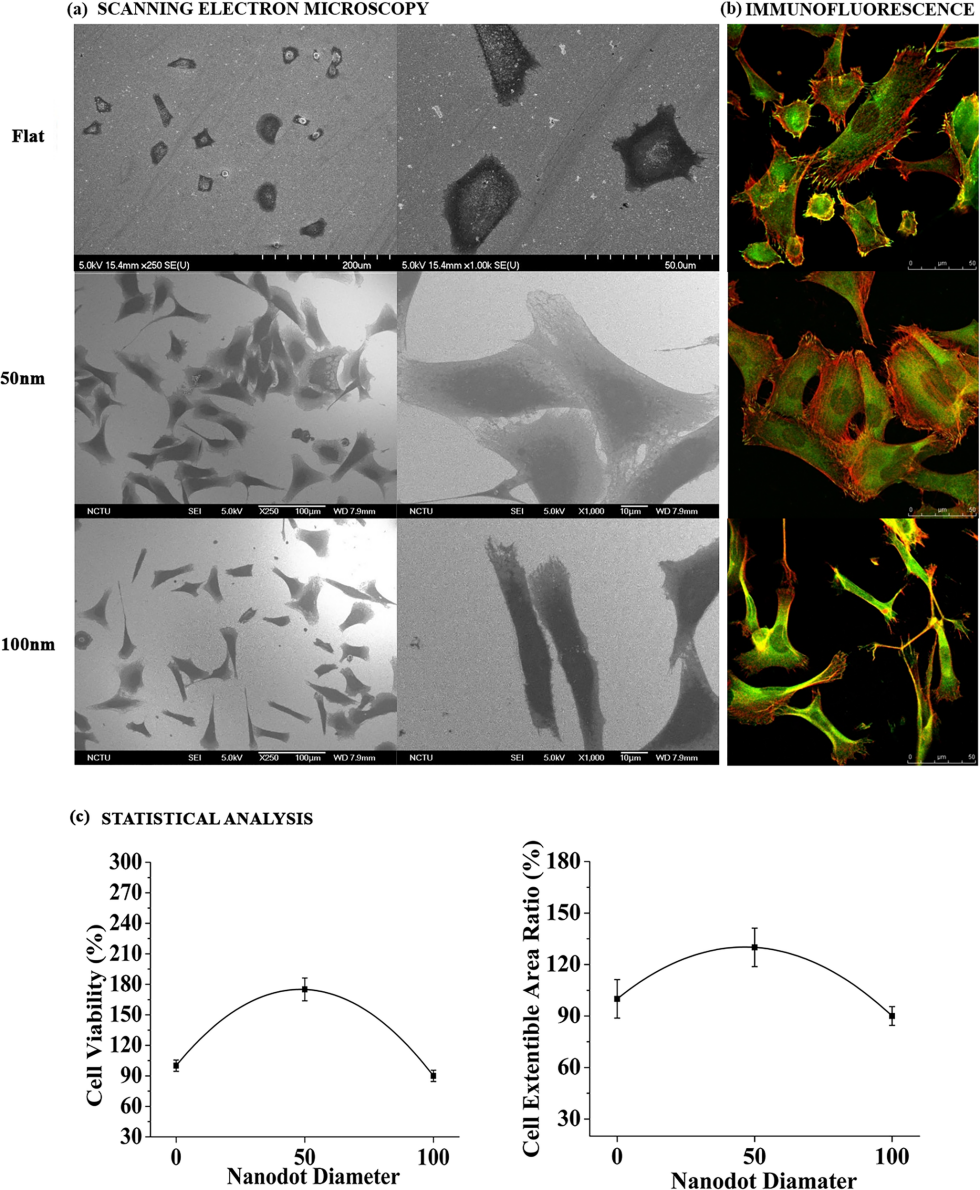
Scanning electron microscopy, Immunofluorescence staining and Statistical analysis of cell area and cell viability of MG63 cells on 50 and 100nm dot diameters. (a): SEM of cells displaying a well extended morphology on the nanodots with 50nm dot diameter in contrast to an elongated morphology on the 100nm dot diameter. Scale bar for Flat = 200, 50μm; for 50nm = 100μm, 10μm; for 100nm = 100μm, 10μm. (b): Immunofluorescence staining of cell cytoskeleton and focal adhesions. Cell morphology was analyzed by staining the cytoskeleton with Phalloidin (Red) and FITC conjugated goat anti rabbit anti-vinculin antibodies (Green). Cells displayed a well extended morphology with a plethora of focal adhesions on nanodots with 50nm dot diameter in contrast to an elongated/constricted morphology with few focal adhesions on the nanodots with 100nm dot diameter. Scale bar = 50μm. (c): Statistical analysis of cell area and cell viability of cells seeded on nanodots with 50 and 100nm dot diameter. Cells displayed a smaller cell area on nanosurfaces with 100nm dot diameter as compared to the nanosurfaces with 50nm dot diameter. Statistical analysis complimented the SEM and Immunofluorescence staining results.

An extended morphology with an excess of focal adhesions to a surface are the hallmark of a healthy cells. Cells with a shrunken morphology and very few focal adhesions represent unhealthy cells which may suffer apoptosis due to Anoikis. Cells were seeded at the density of 1000 cells/cm^2^ for 72 hours on Flat substrates (controls) and nanodots of 2 different diameters (50, 100nm). In the first step, scanning electron microscopy was performed after 72 hours to study the modulation of morphology by the different dot diameters. SEM revealed distinct differences between the cell morphologies on the different nanotopographies. Cells on 50nm nanodot diameter displayed a well extended morphology while the cells on 100nm nanodot diameter displayed a rather elongated/spindle shaped morphology (Fig 3A). The transition in the size of the nanotopographic features caused the modulation in the morphology. In the second step, Immunostaining of cytoskeleton and focal adhesions (Actin and Vinculin, respectively) was performed. Immunostaining of cytoskeleton results complimented the SEM analysis (Fig 3B). Additionally, Immunostaining of focal adhesions (Vinculin) showed that cells on flat (0nm) and 50nm nanodot diameter had more focal adhesions than the cells on 100nm nanodot diameter (Fig 3B). Transition in the number of focal adhesions protruding from cell to the nano-surface by varying the nanotopography size was identified. Also, cells on flat (0nm) and 50nm nanodots displayed vinculin staining on the contact points between cells and the nanodots as well as in the cytoplasm while cells on 100nm nanodots displayed vinculin staining only in the cytoplasm (Fig 3B). Statistical analysis was performed to compare the cell area and cell viability. A 40% increase in the cell area which complimented the well extended morphology was observed on the nanodots with 50nm dot diameter as compared to the cells on the 100nm nanodot diameter (Fig 3C). Accordingly, a 70% increase in the cell viability was also observed on the nanodots with 50nm dot diameter as compared to the 100nm dot diameter (Fig 3C).

### Control of ECM protein interaction with nanodots by the inter-dot spacing and nanodot height

**Fig 4:**
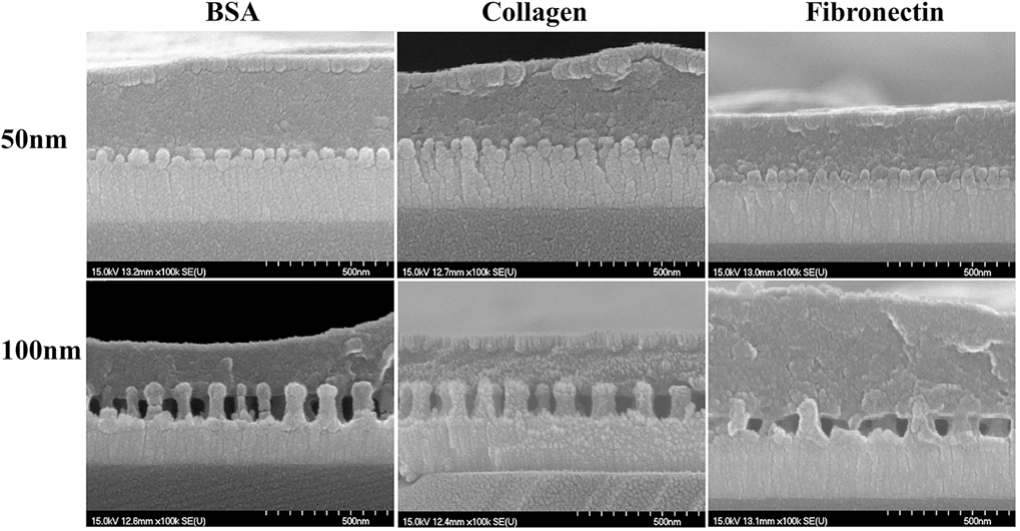
SEM of nanosurfaces coated with BSA, Collagen and Fibronectin. Close interactions of proteins with nanosurface of 50nm dot diameter, 20nm inter-dot spacing and 40nm nanodot height, were seen. Proteins did not appear to interact with base of the nanosurface with 100nm dot diameter due to a greater nanodot height of 100nm and an inter-dot spacing of 70nm. Scale bar = 500nm.

Close interactions between biological entities and the nanotopographies are essential to obtain an improved cell response. To study the reason behind the modulation of cell characteristics by the nanodots, nanodots were coated with three different proteins and nature of interactions between ECM proteins and nanotopographic dimensions (Dot diameter and inter-dot spacing) were analyzed with SEM. SEM analysis revealed the close interactions of the proteins with the 50nm nanodot diameter, 20nm interdot spacing and 40nm nanodot height (Fig 4). However, due to a large inter-dot distance of 70nm between two successive nano-dots and a greater height of 100nm in the 100nm nanodot surfaces, ECM proteins could not interact with the base of the spaces. Hence, the reason behind well-extended morphology with a greater number of focal adhesions on 50nm nanodots (as mentioned in section 3.2) in contrast to an elongated morphology with few focal adhesions on 100nm nanodots, was attributed to the inter-dot spacing between two consecutive nanodots and nanodot height. The ability of the cells to interact with all nanotopographic features of the 50nm nanodots but not with the 100nm nanodots may be hindered by the large inter-dot spacing, as elucidated by SEM of the ECM coated nanodots (Fig 4).

### Inter-dot spacing and nanodot height modulated focal adhesions, induced cell death via Anoikis

**Fig 5:**
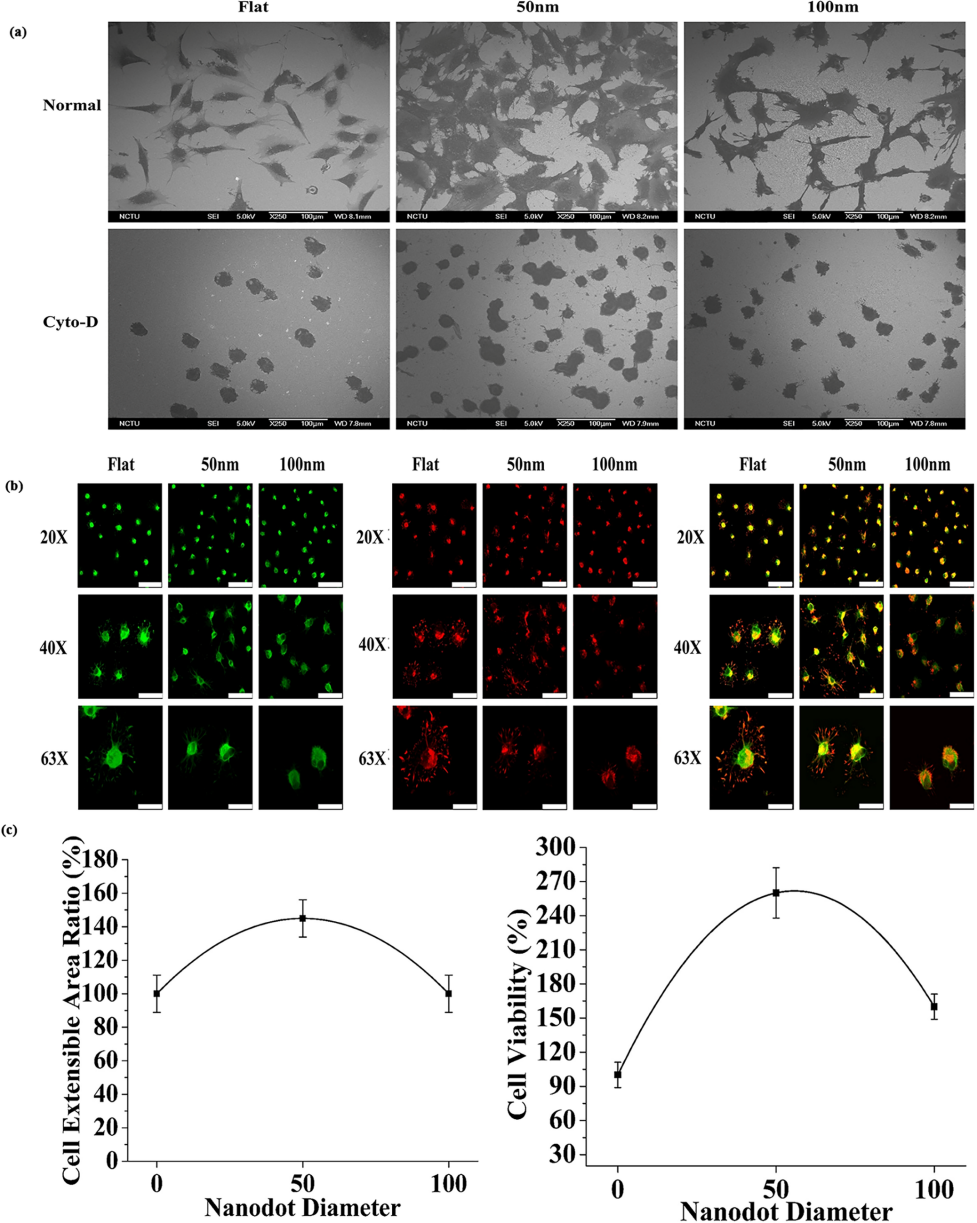
Scanning electron microscopy, Immunofluorescence staining and statistical analysis of cells after Cytochalasin-D treatment on nanodots with different dot diameter. (a): Scanning electron microscopy of cells with/without Cytochalasin-D treatment. Cells displayed a round morphology due to actin de-polymerization by Cytochalasin-D. Very few microfilaments were seen on normal and Cytochalasin-D treated cells showing that even after the morphology transition, cells could not establish adhesions with the nanodots due to a greater nanodot height and inter-dot spacing. Normal represents untreated cells on nanosurfaces. Scale bar = 100μm. (b): Immunofluorescence staining of MG63 cells. From left to right: (i) Vinculin was stained with FITC conjugated goat anti rabbit antibodies (Green). (ii) Cytoskeleton was stained with Phalloidin (Red), and far right displays (iii) Merged images. Scale bars: 20X = 75μm, 40X = 50μm, 63X = 25μm. (c): Statistical analysis of cell area and cell viability of cells seeded on nanodots with 50 and 100nm dot diameter after Cytochalasin-D treatment. Cells displayed a smaller cell area on nanosurfaces with 100nm dot diameter as compared to the nanosurfaces with 50nm dot diameter. Cells also displayed a lower viability on the nanodots with 100nm dot diameter. Cells failed to form focal adhesions with the 100nm nanodots due to a greater height and suffered cell death through Anoikis.

Cells with different morphologies may interact differently with the same nanosurface. Therefore, to study the interactions of cells having a different morphology with our nanosurfaces, cells were treated with Cytochalasin D to cause actin de-polymerization, resulting in a round morphology, to study the nature of interactions of cells having a round morphology with the inter-dot spacing and nanodot height. SEM analysis confirmed actin de-polymerization and transition of morphology from fibroblast-like to round (Fig 5A). Moreover, cells also displayed more microfilaments protruding from the cells on to 50nm microenvironment than on the 100nm (Fig 5A). Cells displayed numerous microfilaments on the 50nm nanodots due to close interactions with a smaller inter-dot spacing of 20nm and nanodot height of 40nm as compared to almost negligible microfilaments on a larger inter-dot spacing of 70nm and nanodot height of 100nm. Immunostaining of cytoskeleton revealed round morphology of the cells after Cytochalasin D treatment (Fig 5B).

Establishment of cellular focal adhesions represents proper cell attachment and is a vital factor governing the cell fate. Lack of focal adhesions can cause cell death (Anoikis). Variation in the number of focal adhesions was observed by varying the inter-dot spacing and nanodot height. Greater number of focal adhesions were seen in cells on the 50nm nanodots due to a smaller inter-dot spacing and nanodot height as compared to negligible focal adhesions on the 100nm nanodots with the 70nm inter-dot spacing and 100nm height (Fig 5B)

Statistical analysis revealed a 45% increase in the cell area on 50nm nanodots with 20nm inter-dot spacing and 40nm nanodot height as compared to the 100nm nanodots with 70nm inter-dot spacing and 100nm nanodot height. Accordingly, a 90% increase in the cell viability was observed on the 50nm nanodots as compared to the 100nm nanodots. The statistics show that cells had favorable interactions with the 50nm nanodots, however, due to larger nanotopographic features of the 100nm nanodots, cells ultimately died due to no favorable interactions (Fig 5C)

### Nanotopography modulated transcription factors and genes related to bone protein secretion

**Fig 6:**
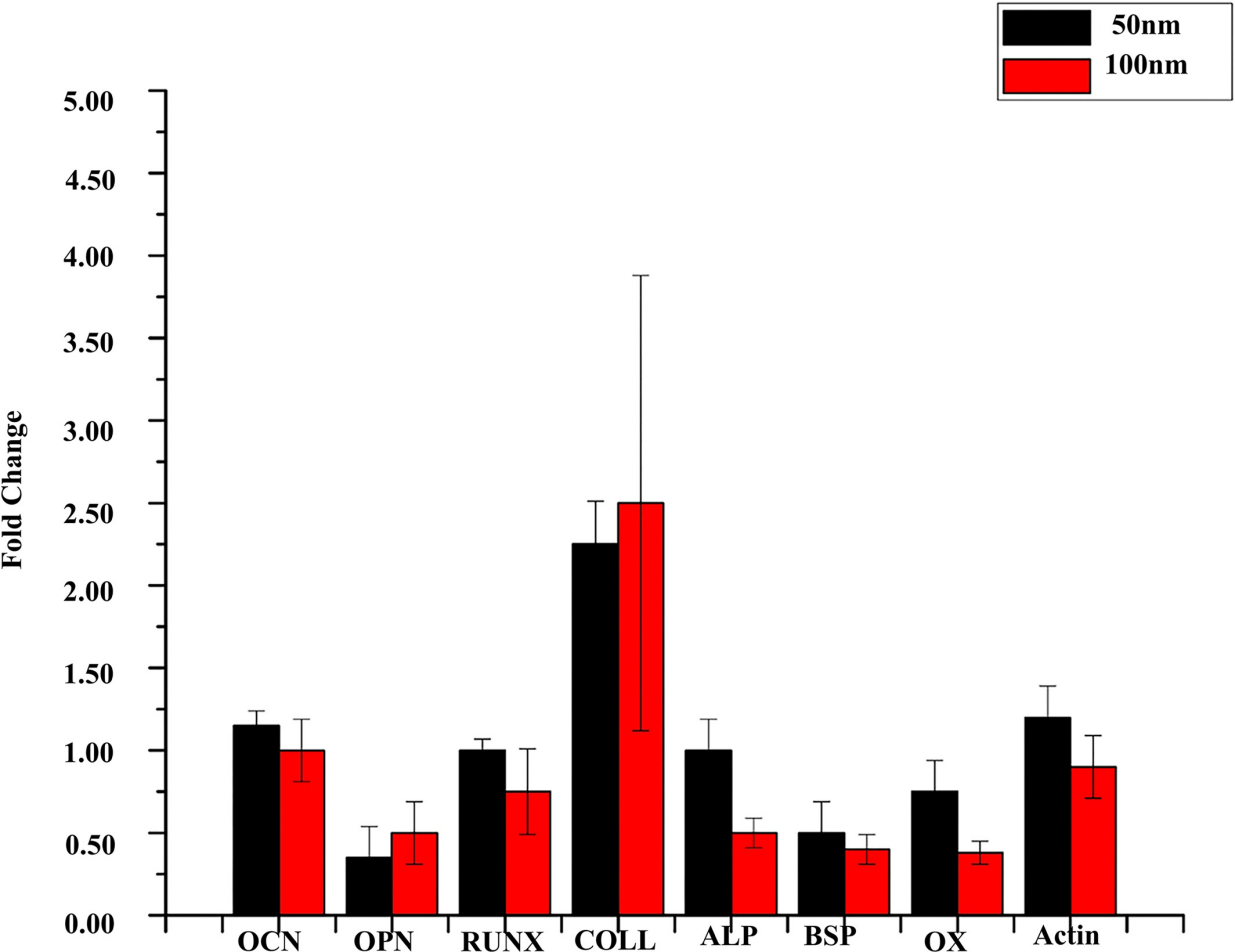
Fold change in expression of transcription factors and genes coding for bone proteins. Fold change in expression of Alkaline phosphatase and Osterix in cells on 50nm dot diameter was twice as compared to fold change in cells on 100nm dot diameter. Additionally, Osteocalcin, RUNX and Bone Sialoprotein were also higher expressed in cells on 50nm nanodots.

It is vitally important that the cells respond to the nanosurface in a way similar to a natural tissue microenvironment, at a genetic level. Therefore, to confirm and identify the transition step in modulating of cell behavior at the genetic level, qRT-PCR was performed to study the regulation of transcription factors, Osterix, RUNX2 and the genes coding for Osteocalcin, Collagen, Alkaline phosphatase and Bone sialoprotein. Transcription factors Osterix and RUNX2 displayed a greater fold change on 50nm nanodots than on the 100nm nanodots (Fig 6). In addition, genes coding for Osteocalcin, Alkaline phosphatase and Bone sialoprotein also showed a greater fold change on the 50nm nanodots than on the 100nm nanodots (Fig 6). Cells on 50nm nanodots with 20nm inter-dot spacing and 40nm nanodot height displayed twice the fold change in Osterix, Alkaline phosphatase as compared to the 100nm nanodots with a 70nm inter-dot spacing and 120nm nanodot height.

## Discussions

Tissue microenvironment displays a highly intricate scenario which consists of cells, ECM, Bio-chemicals [25, 29] which act in a coordinated fashion to guide the cellular behavior and cell fate. Due to the diversity of factors which control the cell behavior, it is a highly challenging task to understand the nature of interactions between the cells and its microenvironment. It has been shown in the past that surface properties like the stiffness of the ECM plays a crucial role in regulating the cellular characteristics such as morphology [30, 31] and focal adhesions. However, since many of the components of this tissue microenvironment lie in the nano-range with which the cells interact [32–34], it is a reasonable assumption that engineering nano-surfaces with dimensions in the range similar to the ECM components may improve the interactions of the cells with the biomaterials.

Given the complexity of the tissue microenvironment, this is not an easy task. However, the first task to solve this puzzle would be to understand the nature of interactions between the cells and its microenvironment. Solving this challenge alone would lead to engineering of nano-surfaces which can favorably regulate cell behavior which would further lead to developments in the fields of Biomedical engineering. The nature of interactions between the cells with its surroundings are a very important factor which needs to be taken into consideration before designing the surface of an implant. In this study, we undertook the challenge to elucidate the regulation of cellular behavior, characteristics and cell fate as a function of nanotopographic features. The in-vivo tissue microenvironment represents 3-Dimensional interactions between the tissue microenvironment and cells. Therefore, while designing the surface of implants, a multi-dimensional approach needs to be considered to trigger a more complimentary response from the cells. Thus, in this study we studied the modulation of cell characteristics and cell fate as a function of not only the nanodot diameter but of inter-dot spacing and the nanodot height.

In our previous study on MG63 cells, we developed the whole range of nanodot arrays from 10 to 200nm to improve the design of the dental implants. We elucidated that 10 and 50nm triggered a favorable cell response. However, the reason for the differential behavior of the cells to the nanotopography was unclear. Moreover, the effect of inter-dot spacing as well as the height of the nanodots in modulating cell behavior was suspected but not analyzed. Therefore, in this study we elucidated the nature of interactions between cells with the different nanotopography features to develop a generalized geometry for the implant surface which can trigger more biocompatible response. Our first task was to investigate if our engineered nano-surfaces (Fig 2), modulated the cell morphology. Our results after seeding the cells for 72 hours clearly demonstrated that both nanotopographies (50, 100nm) caused a morphology modulation in the cells. A well-extended fibroblast-like morphology was displayed by cells on the 50nm nanodots with 20nm inter-dot spacing (Fig 3A) in contrast to an elongated/spindle-shaped morphology on the 100nm microenvironment with 70nm inter-dot spacing (Fig 3A). Thereby, transition in the cell characteristic was observed in response to the different dimensions of the nanodots. One reason for this modulation in the morphology could have been the spacing between two successive nano-structures and nanodot height. Where cells were able to interact with all the dimensions of the nano-structure on the 50nm nanodots due to a smaller inter-dot (20nm) spacing and nanodot height (40nm) and displayed a well extended morphology (Fig 3A), they were only able to interact with the top of the nano-structures on the 100nm microenvironment due to a greater nanodot spacing (70nm) and nanodot height (100nm) which resulted into an elongated/spindle shaped morphology (Fig 3A). This was further clarified by coating the nanosurfaces with ECM proteins which showed close interactions with the 20nm inter-dot spacing and 40nm nanodot height but not with the 70nm inter-dot spacing and 100nm height (Fig 4). Also, our results are consistent with our previous studies on NIH-3T3 [8] and macrophages [2] where we obtained similar response in the morphologies of the cells. This was a very important observation since this implies that even though we changed the cell lines, cells consistently responded to our engineered nanotopographies by displaying similar modulation in the morphology. It also highlights the prospect of using these nanodots on a broader basis for designing different types of implants. Coating of the nanosurfaces with ECM proteins triggered the cells to form excessive focal adhesions (data not shown). This implies that irrespective of the size of the nano-structures, coating of a Biomaterial with proteins can enhance cellular response to the nano-structures. In the next instance we used Cytochalasin D to cause actin de-polymerization [35, 36]. Cells responded by displaying a round morphology (Fig 5A). The number of focal adhesions of cells on the 50nm microenvironment were more as compared to the 100nm microenvironment (Fig 5B). The cells also displayed a greater viability and an extended cell area (Fig 5C) on the 50nm nanodots than the 100nm nanodots. This implied that even if the cells had a morphology which can ease their interactions with the microenvironment, the cells still could not interact with the nanodots due to a greater inter-dot spacing and nanodot height. Moreover, this also indicated that in-order to obtain a more favorable response from the cells, all dimensions of the nanotopographies need to be taken into consideration. Finally, to confirm which nanotopopgraphies triggered a homogeneous and favorable response from the cells, we investigated the modulation of transcription factors and genes responsible for bone protein secretion (Fig 6). Our results were consistent with our previous study. 50nm microenvironment with the inter-dot distance of 20nm triggered the cells to respond in a more favorable way as compared to the nanodots with 100nm diameter and 70nm inter-dot spacing.

The findings of this study may be limited to osteoblasts, however this opens new doors towards designing nano-surfaces with improved geometries for implants. Besides, the consistency in the findings of this study with our previous studies also highlights the prospects of using Tantalum oxide to engineer implants/prosthetics for different body parts. Other studies reporting the cell response to different nanotopographies reported enhanced cytoskeletal organization, cell growth and cell proliferation on nano-islands of size smaller than 27nm [37]. However, size greater than 80nm hindered the cell growth. Tamplenizza et al. studied Titanium dioxide in inducing differentiation in PC12 cells [38]. We have also shown in our previous studies that nano-structures of size smaller than 50nm displayed more control over the focal adhesion regulation by the integrins through the ERK^1/2^ pathway [4]. We have also studied the regulation of cell behavior on the genetic level in our previous study where cells responded to the 50nm nano-dots by secreting more Nitric oxide than the rest [5]. Furthermore, many studies have shown the temporal regulation of cell response to the nano-structures [39]. Therefore, it is important to study the behavior of the cells on any nano-structure as a function of time before exploiting its use as an implant material. Tantalum metal has also been exploited as the material to engineer bone, dental implant [40–42] and also as a radiographic marker [43]. Moreover, once an implant geometry is experimentally verified to be apt for a particular application, various methodologies such as Nanoimprint lithography (NIL) as shown by Bergmair et al. [44] or by indirect 3-Dimensional printing methods as demonstrated by Tamjid et al. [45] can be employed to generate uniform nanotopographies of interest for improved bio-nanosurface interactions. The findings of this study will prove to be highly beneficial in designing the surface of the implants. In extension, the results of this study can also be exploited in designing improved nano-surfaces of other materials apart from Tantalum to be used as implants with improved geometries.

All of these results collectively demonstrate how the precise control of all dimensions of the nano-topographies can entice favorable cellular response towards a Biomaterial. This study may also open new doors towards engineering biomaterials with new and improved nanotopograhies with size in range of tissue microenvironment components which the body may recognize as “self” and may decrease instances of immune response. In general, the findings of this study will be highly beneficial in the implant’ surface design and in the fields of Biomedical and tissue engineering.

## Conclusions

In this study we evaluated the effects of inter-dot spacing, nanodot height and the nanodot diameter on the cell behavior, characteristics such as cell morphology, cell area, focal adhesions, secretion of bone proteins and ultimately the cell fate in osteoblast cell line MG63. Thus, the nanodot diameter, inter-dot spacing and the nanodot height were used as parameters to propose the design of the nanosurfaces which may trigger a favorable cell-response. We showed that the inter-dot spacing along with the nanodot height played a crucial role in governing the cell behavior and cell fate. Cells failed to interact efficiently with the nanodots having 100nm dot diameter, 100nm nanodot height and 70nm inter-dot spacing which resulted in cell death via Anoikis. Thus, we engineered nanosurfaces with improved nanotopographic features for a favorable cell response.

Due to the limitation of the fabrication set up, more experiments may be required to elucidate the effect of other parameters such as nanotopography shape on the cell fate. According to the results of this study, Inter-dot spacing of ≤ 20nm and nanotopographic heights of ≤ 40nm with a dot diameter of 50nm may provide an improved environment for the cells to attach and grow. Hence, the inter-dot spacing and nanodot height is an important factor which needs to be considered by the biomedical engineers while designing the surface of an implant. Applications of the findings of this study may are expected in the fields of Tissue and Biomedical engineering.
